# A Qualitative Survey of Five Antibiotics in a Water Treatment Plant in Central Plateau of Iran

**DOI:** 10.1155/2013/351528

**Published:** 2013-04-11

**Authors:** Mohsen Heidari, Maryam Kazemipour, Bijan Bina, Afshin Ebrahimi, Mehdi Ansari, Mohammad Ghasemian, Mohammad Mehdi Amin

**Affiliations:** ^1^Environment Research Center, Isfahan University of Medical Sciences, Isfahan 81746-73461, Iran; ^2^Department of Chemistry, Faculty of Sciences, Islamic Azad University, Kerman Branch, Kerman, Iran; ^3^Pharmaceutics Research Center, Faculty of Pharmacy, Kerman University of Medical Sciences, Kerman, Iran

## Abstract

*Introduction*. This study aimed to survey a total of five common human and veterinary antibiotics based on SPE-LC-MS-MS technology in a water treatment plant at central plateau of Iran. Also two sampling techniques, passive and grab samplings, were compared in the detection of selected antibiotics. *Materials and Methods*. In January to March 2012, grab and passive samples were taken from the influent and effluent of a water treatment plant. The samples were prepared using solid-phase extraction (SPE), and extracts were analyzed by liquid chromatography tandem mass spectrometry (LC-MS-MS). *Results*. The results showed that enrofloxacin, oxytetracycline, and tylosin were not detected in none of the samples. However, ampicillin was detected in the grab and passive samples taken from the influent (source water) of the plant, and ciprofloxacin was detected in passive samples taken from the influent and effluent (finished water) of the plant. *Conclusion*. The results imply that passive sampling is a better approach than grab sampling for the investigation of antibiotics in aquatic environments. The presence of ampicillin and ciprofloxacin in source water and finished water of the water treatment plant may lead to potential emergence of resistant bacteria that should be considered in future studies.

## 1. Introduction 

Pharmaceuticals are used extensively in human and veterinary medicine [1]. More than 3000 different chemical substances are used as human medicines and in farming and aquaculture applications, in which antibiotic is one of the most important groups of common pharmaceuticals in our daily lives [2]. Besides the critical role of antibiotics in human health, they are potential environmental contaminants, so that there has been increasing concern within the scientific community regarding the presence of different types of drugs in the environment since the second half of the 1990s [3]. 

There are different pathways for releasing of antibiotics to the aquatic environment. After the administration to humans, their metabolites along with noneliminated parent compounds are excreted into the sewage [4]. Wastewater treatment plants (WWTPs) are not designed to completely remove antibiotics, and consequently they are released into natural waters. Moreover, antibiotics can pass through all natural filtrations and reach ultimately to drinking water due to their high water solubility and often poor degradability [5]. Furthermore, antibiotics are extensively used in fish farms, in which they are used as feed additives or they are directly applied into the water. The result of an overfeeding of these compounds to the fish farm is that many compounds end up in the sediments where they are slowly degraded or slowly leach out back into the surrounding waters [4]. Use of veterinary antibiotics for the treatment of bacterial infections of animals as well as prophylactic agents is another source of contamination. The animal excretion is the major route of contamination, as the most of these substances end up in manure. The parent compounds or their biologically active metabolites present in the manure may move from the field to the groundwater and eventually enter surface waters through events of rain [3, 4].

Little is known about possible human and ecological adverse effects of antibiotics in the aquatic environment. Although the concentration levels of these compounds seem not to cause toxic effects on human health and in the aquatic environment, there is a big concern on the long-term and continuous exposure of aquatic organisms to them [1, 6]. Low levels of antibiotics have been linked to the increased emergence of resistant strains of pathogenic bacteria that have potential to impact human health. In addition, a cross-resistance can be developed between antibiotics used in veterinary medicine with those of similar structures used exclusively in human medicine [7, 8]. Therefore, the occurrence of antibiotics in the environment has received considerable attention.

The analysis of antibiotics in the environment represents a difficult task due to the high complexity of the matrices analyzed and to the usually low concentrations at which target compounds are present in the aquatic environments. This condition leads to the development of very sensitive analytical methods suitable for the monitoring of these analytes in low concentration levels [4]. However, the most common approach for the analysis of antibiotics in environment includes a preconcentration step by solid-phase extraction (SPE) and a liquid chromatographic separation coupled with mass spectrometry (LC-MS) or tandem mass spectrometry (LC-MS-MS). Thus, SPE-LC-MS (MS) methods are capable of separation and qualitative and quantitative detection of antibiotics with low detection limits [1, 12].

For surveying of antibiotics in aquatic environment, traditional water-column sampling (including grab and composite samplings) is usually used. However this methodology has many shortcomings, including insufficient water sampled to satisfy the detection limit requirement of analytical methods, lack of time-weighted average (TWA) of pollutants level in aquatic media, and physical and financial difficulty for repetitive sampling. Time-integrative passive sampling, in contrast to grab or composite sampling, enables estimates of TWA contaminant concentration over extended sampling periods [13, 14]. In this way, the preconcentration of contaminants leads to an increase in the capability for detecting trace concentrations. Antibiotics similar to others pharmaceutics and polar pesticides could be sampled from water by Polar Organic Chemical Integrative Sampler (POCIS) [15]. The subsequent laboratory procedure (i.e., extraction, identification, and determination of analytes) in POCIS is the same as in the case of traditional sampling techniques [16].

Up to now, numerous studies have been done on the occurrence of antibiotics in various compartments of the aquatic environment, for example, municipal wastewater, industrial wastewater, hospital wastewater, surface water, ground water, and drinking water as well [17–21]. Also, in recent decade, detection of antibiotics in aquatic environment through passive sampling followed by SPE and LC-MS (MS) has received considerable attention [14, 15]. The aim of the present paper is to analyze a total of five common human and veterinary antibiotics, selected from four important categories, including quinolones (ciprofloxacin and enrofloxacin), macrolide (tylosin), *β*-lactam (ampicillin), and tetracycline (oxytetracycline) based on SPE-LC-MS-MS technology in a water treatment plant at central plateau of Iran. In this study, we compared the Polar Organic Chemical Integrative Sampler (POCIS) as a passive sampler to standard grab sampling technique for the detection of selected antibiotics.

## 2. Experimental

### 2.1. Chemicals and Materials

**Figure 1:**
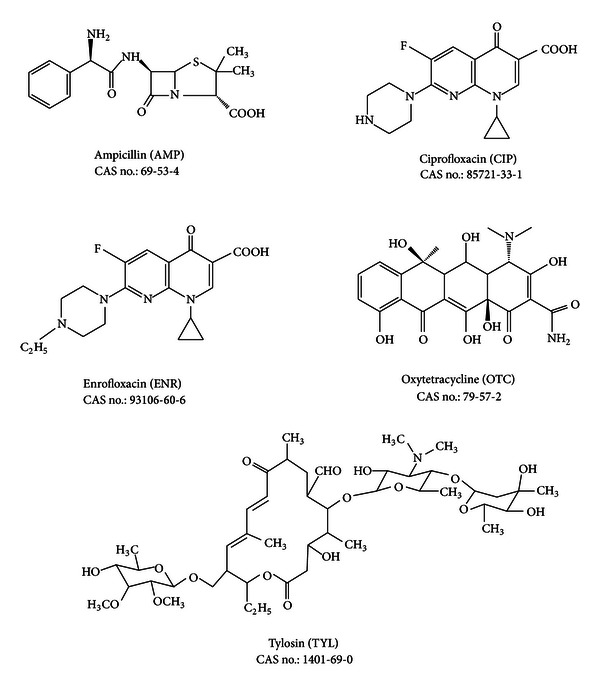
Chemical structures of the antibiotics investigated.

Five antibiotic standards including ampicillin (analytical standard), ciprofloxacin (≥98% purity), enrofloxacin (≥98% purity), oxytetracycline (≥95% purity), and tylosin (analytical standard) were purchased from Sigma-Aldrich (Germany). Structures of the investigated compounds are shown in Figure 1. Also some physicochemical properties of the investigated antibiotic compound are described in Table 1. HPLC grade methanol and ultrapure water were purchased from Merck (Darmstadt, Germany). Oasis hydrophilic-lipophilic balance (HLB) cartridges (200 mg/6 mL) were purchased from Waters (Milford, MA, USA). 0.45 *μ*m cellulose acetate filter and 0.2 *μ*m cellulose acetate syringe filter were the products of Millipore (USA) and Whatman (Diesel, Germany). The following chemicals were all in analytical grade: sulfuric acid (purity 99%) from Fluka and disodium ethylenediamine tetraacetate (Na_2_EDTA) and sodium thiosulfate (Na_2_S_2_O_3_) from Sigma-Aldrich.

Individual stock solution for each antibiotic was prepared in the mixture (1 : 1, volume : volume) of MeOH and high-purity water at a concentration of 0.05 to 0.5 mg/mL and stored in a freezer (−10°C). Working standard mixture solutions (0.02 to 5 *μ*g/mL) were made by diluting the stock solutions with the mixture of MeOH and high-purity water (3 : 1, v : v) every time just before use and storing at 4°C. All standard solutions (including stock and working solutions) were stored in glass bottles covered by aluminium foil at −10°C in a freezer. All glassware was washed with detergent and hot water, rinsed with distilled water and acetone, and dried in the oven at 220°C overnight.

### 2.2. Grab Sampling

#### 2.2.1. Sample Collection and Preparation

**Figure 2:**
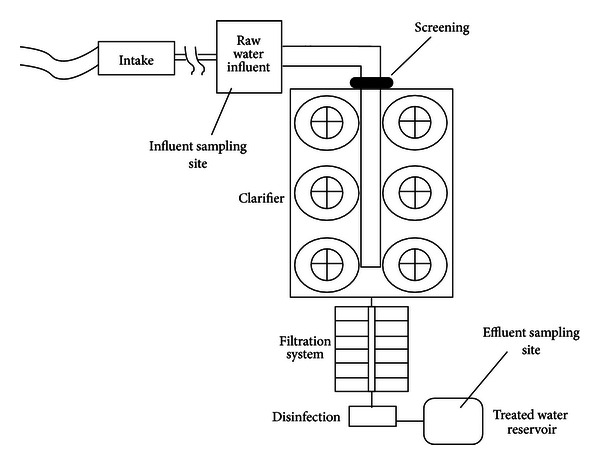
Schematic design of the WTP and sampling sites.

From January to February 2012, grab water samples (from each site on the first and the last day of the POCISs exposure period) were taken from two locations of a water treatment plant. Source water samples were collected at the plant intake prior to any water treatment process, and finished water samples were collected at the reservoir of treated water. A schematic design of the WTP and sampling sites is shown in Figure 2. The plant has a 12.5 m^3^s^−1^ capacity and is fed by a perennial river in the central plateau of Iran. The river flows through a region with medium population density and high agriculture and aquaculture activities.

Water samples were collected in 2.5 l amber glass bottles with screw cap. Before sampling, the bottles were cleaned following the procedure previously described. For finished water samples, excess quenching agent (sodium thiosulfate) was added to dechlorinate the sample. The glass bottles containing samples were shipped to laboratory under cool conditions before further treatment and analysis. In laboratory, water samples were filtered through a 0.45 *μ*m acetate cellulose filter and were acidified by adding 3.0 M H_2_SO_4_, followed by addition of 0.2 g disodium ethylenediamine tetraacetate (Na_2_EDTA). Under such conditions any antibiotic activity in the samples was kept to the minimum, and their tendency to be bound to divalent ions may be decreased. The samples were stored in dark at 4°C until they were extracted, typically within 1 week.

#### 2.2.2. Solid-Phase Extraction

**Figure 3:**
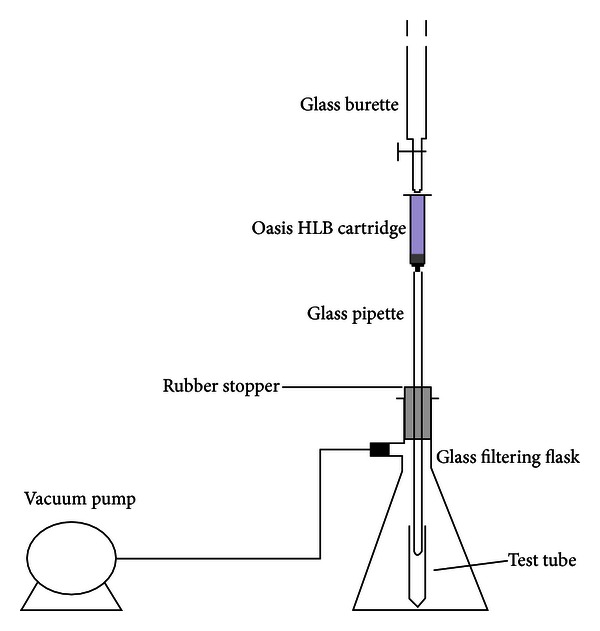
Schematic of SPE set up.

Solid-phase extraction (SPE) experiments were conducted using 200 mg/6 mL Oasis HLB cartridges on an innovative setup (Figure 3). The cartridges were preconditioned with 4 mL of MeOH and 6 mL of deionized water. A volume of 1000 mL of water sample with pH 2.8–3 (H_2_SO_4_) was passed through the cartridge at a flow-rate of 5–8 mL min^−1^ using a vacuum extraction manifold at 7–9 in.Hg (Visiprepä, Supelco, Bellefonte, PA, USA; 1 in.Hg = 338.638 Pa). Afterwards the cartridges were rinsed with 10 mL of ultra-pure water and were air-dried for 5 min. The retained analytes were subsequently eluted with 10 mL of methanol into a glass test tube. The extract was concentrated to dryness under a stream of *N* and reconstituted to ~250 *μ*L in a solvent mixture of ultra-pure water/methanol (9 : 1). The extract was filtered through a 4 mm i.d., 0.2 *μ*m pore size cellulose acetate syringe filters, transferred to an amber vial, and stored at −15°C until LC-MS/MS analysis. 

### 2.3. Passive Sampling

#### 2.3.1. POCISs Characterization

Polar Organic Chemical Integrative Samplers (POCISs) consist in a sequestration medium, such as HLB, enclosed within two hydrophilic micro-porous polyethersulfone membranes for the integrative sampling of polar organic chemicals such as antibiotics. A detailed description of this sampling technology and its sorbent material is described by Alvarez et al. [22]. In this study, the POCIS discs had a standard configuration, that is, 180 cm^2^ sampling surface area per gram of sorbent [22]. 

#### 2.3.2. Field Deployment of POCISs

**Figure 4:**
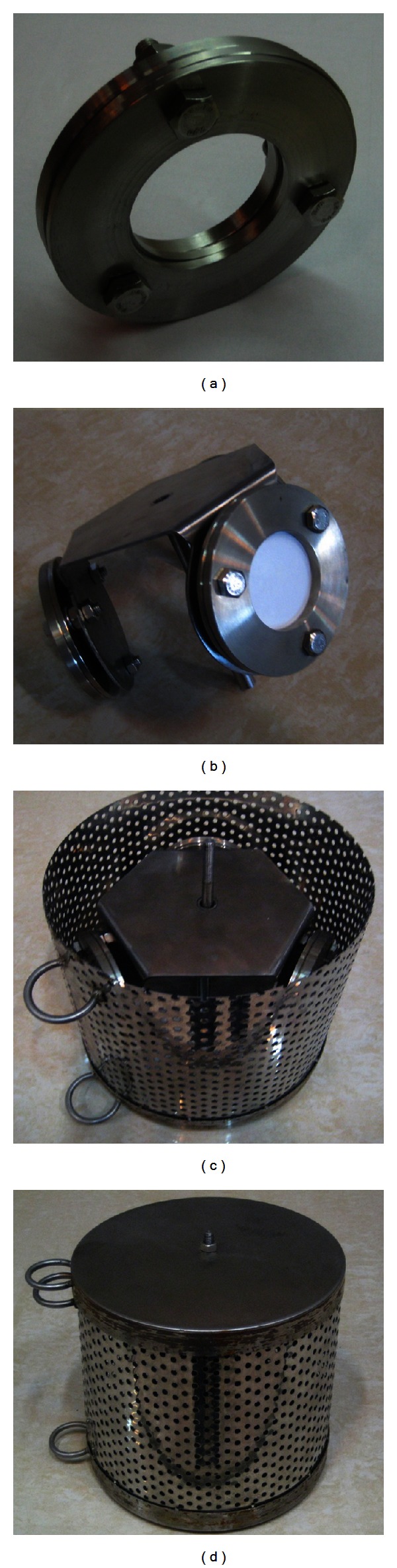
The used POCISs and deployment steel canister.

The POCISs samples were placed in the same location and time as the grab sample was collected. At each site, a protective steel canister containing three POCISs, each with approximately 39.2 cm^2^ of effective sampling surface area, was deployed for 30 days (from January to February 2012).  Figure 4 shows the POCIS and deployment steel canister. Before deployment, the sorbent, HLB, was preconditioned with 6 mL of MeOH followed by 10 mL of HPLC-grade water and left at room temperature until dry. 

The canisters were in a vertical position and at a depth of 2 m in the water column. At the end of the exposure period, the POCISs were collected, rinsed with water, kept in the containers, and transported to the laboratory under cooled conditions. Upon reception, the POCISs were stored frozen before extraction. 

#### 2.3.3. Recovery of Chemical Residues from POCIS

Procedures for the recovery of the sequestered chemical residues from the deployed POCISs are described in detail by Bueno et al. [15]. Briefly, the POCISs were disassembled, and the HLB sorbent was transferred into empty SPE cartridges (6 cm^3^) and packed between two polyethylene frits. The analytes from the sorbent were eluted with 15 mL of MeOH at 1 mL/min into a glass test tube. At the last step, the eluate was evaporated until almost dryness under a gentle stream of nitrogen at 35°C and reconstituted in 250 *μ*L in a solvent mixture of ultrapure water/methanol (9 : 1). The extract was filtered directly into an analysis vial using a 0.2 *μ*m cellulose acetate syringe filters, ampoulated, and stored at −15°C until LC-MS/MS analysis. In order to increase the total mass of sequestered residues, each ampoulated sample was a composite of three individual POCIS extracts from the same deployment canister.

### 2.4. LC-MS-MS Analysis

The extracts were separated on the reverse phase Zorbax Eclipse XDB-C18 column, 4.6 mm × 50 mm ID and 1.8 *μ*m particle size (Agilent Technologies, CA, USA) using LC system with a quaternary pump, a vacuum degasser, and an autosampler. The injection volume of sample aliquots was 5 *μ*L, and a binary gradient with a flow rate of 0.5 mL/min was used. Mobile phase A contained 0.1% aqueous solution of formic acid (v/v) and mobile phase B contained 0.1% formic acid (v/v), in meOH. The gradient started with 0% of mobile phase B for 0.5 min, increased to 20% from 0.5–3 min, to 70% from 3.0–7.5 min, and to 95% from 7.5–11 min, decreased to 0% from 11-12 min, and remained at 0%. All target compounds were eluted out of the column within 15 min, and the autosampler was operated at room temperature.

The flow from the LC column was transferred to a triple-quadrupole mass spectrometer equipped with an ESI source. The electrospray voltage was 4 kV, the capillary temperature 350°C, and maximum isolation time 200 ms. Nitrogen was used as the nebulising and drying gas, and a nebulizer pressure of 20 psi and a drying gas flow of 13 L/min were selected. 

## 3. Results

The results of this study include optimal instrumental conditions for analysis of subjected antibiotics, representative MS/MS spectra for the analytes and occurrence of the antibiotics in water samples. 

The optimized LC-MS/MS parameters and the information of calibration curves are summarized in Table 2. Because all antibiotics belong to groups 1 and 2 EPA Pharmaceutical compounds [23], they were separated in ESI+.

**Figure 5:**
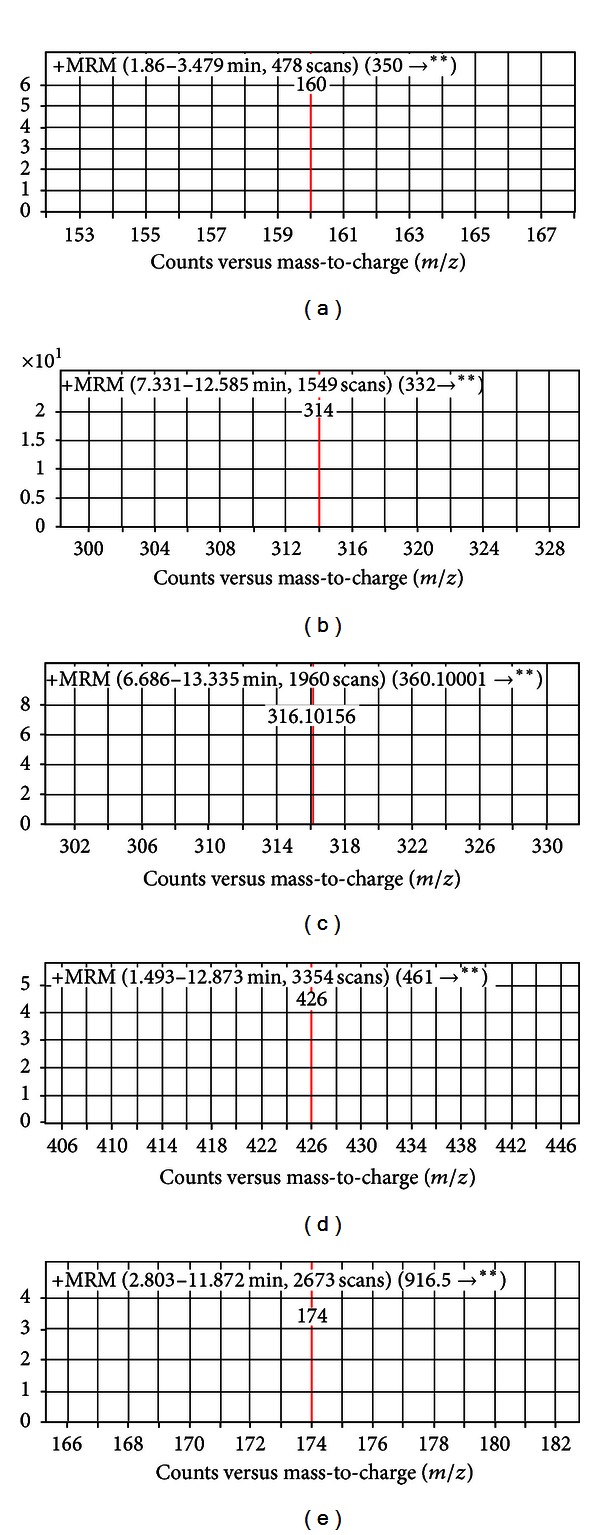
The MS/MS spectra obtained for (a) AMP, (b) CIP, (c) ENR, (d) OTC, and (e) TYL in standard solutions.

Figure 5 shows representative MS/MS spectra obtained for the antibiotics in standard solutions. The figures represent product *m*/*z* data obtained for the analytes.

Two of 5 antibiotics were detected in the analyzed samples of raw and treated water at the WTP (Table 3). Ampicillin was detected with LC-MS/MS for both grab and passive samples at influent sampling site; however ciprofloxacin was detected only for passive sample. Other analytes that are ENR, OTC, and TYL were not detected by any of sampling procedures. From all samples taken from effluent sampling site, we could detect CIP through passive sampling SPE-LC-MS/MS. 

## 4. Discussion

In this study, the occurrence of five antibiotics was investigated qualitatively in raw and treated water at a water treatment plant in central plateau of Iran. Our primary aim was to investigate the occurrence of the antibiotics quantitatively. Thus calibration curves for each analyte were set, and their correlation coefficient were >0.99 (Table 2). However because of some limitations such as lack of valid recovery and matrix effect data, and economical and technical restrictions, we decide to report the results as present/absent.

Analyzing very low levels of analytes in aqueous environments requires optimal sampling, processing, and analyzing conditions [4]. In order to prevent glassware contamination, they were conditioned according to the literature, namely, repeatedly washing, rinsing, and baking [23]. In grab sampling, adding sodium thiosulfate to finished water samples, acidifying all samples, and storing them at low temperatures and in dark ambient all were necessary to avoid decomposition of analytes by means of chemical reactions and microbial activity [4]. In accordance with the literature in this field, a chelating agent, namely, Na_2_EDTA, was applied to decrease the tendency for antibiotics to bind to metals or multivalent cations in the matrix, to improve peak shape, and to prevent interferences during the extraction of antibiotics [4, 24]. 

Solid-phase extraction (SPE) arrangement was nearly according to EPA Method 1694 [23]. There are some suitable cartridges for extraction of antibiotics from aqueous matrixes; however the most common SPE cartridge is hydrophilic-lipophilic balance (HLB) [25]. So we use 200 mg/6 mL Oasis HLB cartridges in an innovative extraction setup (Figure 3). Sample pH and eluant were proved to be crucial parameters for antibiotics preconcentration using SPE (14). Solution pH is expected to significantly influence speciation of the antibiotics owing to the presence of acidic and basic functional groups in their structures (Figure 1). Their acidity constants (Table 1) indicate that protonation and deprotonation of these antibiotics occur readily in the environmental pH range [26]. Acidifying samples to pH 2.5–3 was done, because the selected antibiotics belong to groups 1 and 2 EPA Pharmaceutical compounds (with acidic nature), and acidic condition leads to better recovery of them from the aqueous matrix [23]. Tong et al. reported that, at pH 2.0, recoveries of FQs and TCs were more than 70% and 60%, respectively, whereas under neutral condition, those of TCs and FQs were less than 30% [27]. Reverté et al. selected pH 2.8 for sample conditioning before SPE of TCs and Qs from river water samples [28]. 

According to EPA Method 1694 [23], ESI (+) mode was selected for separation of the analytes by LC. Chromatographic separation was optimized with a series of preliminary experiments, utilizing various mobile phases consisting of MeOH, formic acid, and water at various fractions. The MeOH was selected as it was commonly used as organic mobile phase in LC-MS/MS system [29, 30]. Addition of formic acid into mobile phase can affect the chromatographic separation, change the pH value of mobile phase, and affect ionization efficiency [31]. The formic acid in various concentrations in both mobile phases A and B was evaluated for the optimal chromatographic separation, and 0.1% acid formic was added to both mobile phases. Column temperatures of 25 [32], 30°C [33], and room temperatures [30] were widely applied to LC-MS/MS for selected antibiotics detection. In this study, the column was operated at room temperature. Elution with identical gradient conditions at different flow rates showed that the optimal flow rate was 0.5 mL min^−1^.

The surveyed antibiotics belonged to fluoroquinolone (CIP and ENR), tetracycline (OTC), macrolide (TYL), and *β*-lactams (AMP). According to Table 3, two of all five antibiotics were detected in raw water introduced to the water treatment plant (AMO and CIP). Moreover, CIP was detected in finished water through passive sampling. The water of the plant is served by a perennial river, which flows through a region with medium population density and high aquaculture activities. The river drainage area is subjected to pollution from several point and nonpoint sources. There are one city with more than 20000 populations and several small towns and villages in upstream of the source water sampling point (WTP) in which some households and industries discharge illegally their wastewater into the main drain in the vicinity or to the river. Also there is an important fish farming area in upstream which is supplied by the river water. Therefore, the occurrence of AMP (with veterinary and human use) and CIP (human use) may possibly be explained by illegal discharges from aquaculture farms and residential areas in addition to runoff from agricultural fields located on the river banks upstream of sampling point (i.e., entrance of the water treatment plant). Ampicillin, like other *β*-lactam antibiotics, due to the chemically unstable *β*-lactam ring, readily undergoes hydrolysis [4]. Therefore, it was expectable that ampicillin was not detected in finished water.

The presence of antibiotics in aqueous environments is a matter of concern because of possible development of resistant strains of bacteria. Accordingly, there are some reports about prevalence of ampicillin- and ciprofloxacin-resistant bacteria in river waters, water treatment plants, and drinking waters [34–36]. Therefore the presence of some antibiotics in source and finished water of the subjected water treatment plant is of concern, especially in view of potential emergence of resistant bacterial strains in drinking water that is served for about 4 million people. This investigation highlights the need for surveying multiantimicrobial-resistant bacteria (at least for AMP and CIP) in the finished water of the water treatment plant and its source water.

Another important message from this study is that passive sampling (or POCIS) was more efficient than grab sampling, at least qualitatively, in monitoring of the antibiotics in water environment. As can be seen from Table 3, among two sampling points and five antibiotics to be monitored, we could detect only AMP in source water through grab sampling. On the other hand, AMP and CIP in source water and CIP in finished water were detected by SPE-LC-MS-MS through passive sampling technique. This finding is in accordance with those found by Alvarez et al. [22], who showed that passive sampling could detect more organic contaminant including antibiotics than water-column sampling in aqueous environments. The reason for this is that POCIS monitors the trace contaminants over extended periods of time, for example, 30 days in our study. This feature permits preconcentration of contaminants and sequestration of residues from episodic events commonly not detected with grab sampling. In fact, by using passive sampling technique, regular monitoring of the antibiotics can serve to track both spatial and temporal trends in waters [15]. Generally, POCIS similar to other passive samplers is typically easier to handle, preserve, and transport than water samples comprised of several liters. Thus, the POCIS provides an increase in method sensitivity and simplicity in use.

## 5. Conclusion

An SPE-LC-MS/MS single residue method was used for the survey of 5 antibiotics in source water and finished water of a water treatment plant (WTP) in central plateau of Iran. The water samples were collected by two sampling techniques, that is, grab and passive samplings. Because of some technical and economical limitations and the lack of valid recovery and matrix effect data, the presence of the antibiotic was assessed qualitatively. The results of this study showed that ciprofloxacin and ampicillin were detected in source water, and ciprofloxacin was detected in finished water. Based on the findings, it was implied that POCIS was more efficient than grab sampling in detection of the antibiotic in water environment. The presence of AMP and CIP in water of investigated WTP may lead to potential emergence of resistant bacteria that should be considered in future studies. Finally, the implications of our findings may not be straightforward in relation to public health; nevertheless, our study does highlight the need for more extensive investigation on the occurrence of antimicrobial compounds and bacterial resistance to them in surface waters in Iran.

## Figures and Tables

**Table 1:** Physicochemical properties of the investigated antibiotic compounds.

Compound	Formula	MW	Antibiotic class	p*K*_*a*_
AMP	C_16_H_19_N_3_O_4_S	349.4	*Β*-lactam	2.7, 7.3 [9]
CIP	C_19_H_22_FN_3_O_3_	331.3	Fluoroquinolone	3.01, 6.14 [10]
ENR	C_17_H_18_FN_3_O_3_	359.4	Fluoroquinolone	3.85, 6.19 [10]
OTC	C_22_H_24_N_2_O_9_	460.5	Tetracycline	3.22, 7.46 [10]
TYL	C_46_H_77_NO_17_	916.1	Macrolide	7.5 [10], 7.1 [11]

**Table 2:** Optimal conditions for the analysis of selected antibiotics and related calibration curves.

Compound	ESI	Time segment (min)	*m*/*z* parent ion	*m*/*z* daughter ion	Collision energy (eV)	Fragmentation amplitude (V)	Calibration curves	
							Equation, *n* ^a^	*R* ^2^
AMP	+	1.86–3.48	350	160	20	90	*y* = 410*x* − 30, 3	0.998
CIP	+	7.33–12.58	332	314	20	110	*y* = 927*x* + 2640, 3	0.993
ENR	+	6.69–13.33	360	316	20	90	*y* = 211*x* + 1437, 3	0.999
OTC	+	1.49–12.87	461	426	20	90	*y* = 78*x* + 71, 3	0.998
TYL	+	2.80–11.87	916	174	35	110	*y* = 709*x* + 697, 3	0.998

^a^Number of concentrations for plotting calibration curves.

**Table 3:** Occurrence of investigated antibiotics in the subjected water treatment plant.

Compound	Influent sampling site		Effluent sampling site	
	Grab sampling	Passive sampling	Grab sampling	Passive sampling
Ampicillin	Detected	Detected	ND	ND
Ciprofloxacin	ND^1^	Detected	ND	Detected
Enrofloxacin	ND	ND	ND	ND
Oxytetracycline	ND	ND	ND	ND
Tylosin	ND	ND	ND	ND

^1^Nondetected.
